# Drug–Drug Interactions of Infectious Disease Treatments in Low‐Income Countries: A Neglected Topic?

**DOI:** 10.1002/cpt.1397

**Published:** 2019-03-21

**Authors:** Savannah J. McFeely, Jingjing Yu, Ping Zhao, Susan Hershenson, Steven Kern, Isabelle Ragueneau‐Majlessi, Dan Hartman

**Affiliations:** ^1^ School of Pharmacy University of Washington Seattle Washington USA; ^2^ The Bill & Melinda Gates Foundation Seattle Washington USA

## Abstract

Despite recent advances in recognizing and reducing the risk of drug–drug interactions (DDIs) in developed countries, there are still significant challenges in managing DDIs in low‐income countries (LICs) worldwide. In the treatment of major infectious diseases in these regions, multiple factors contribute to ineffective management of DDIs that lead to loss of efficacy or increased risk of adverse events to patients. Some of these difficulties, however, can be overcome. This review aims to evaluate the inherent complexities of DDI management in LICs from pharmacological standpoints and illustrate the unique barriers to effective management of DDIs, such as the challenges of co‐infection and treatment settings. A better understanding of comprehensive drug‐related properties, population‐specific attributes, such as physiological changes associated with infectious diseases, and the use of modeling and simulation techniques are discussed, as they can facilitate the implementation of optimal treatments for infectious diseases at the individual patient level.

Contrary to developed countries, major infectious diseases, such as HIV/AIDS, malaria, and tuberculosis (TB), continue to cause the majority of deaths in low‐income countries (LICs) worldwide.^1^, ^2^ Even when effective treatment options exist, poor understanding of what constitutes safe and effective use of these medications leads to adverse drug reactions or loss of efficacy, with the later contributing to drug resistance. An overarching risk factor is ineffective management of drug–drug interactions (DDIs) that can lead to changed systemic drug exposure, resulting in variations in drug response of the coadministered drugs.^3^ Recognizing the significance of DDIs, leading regulators in the world require assessment and management of DDIs as an integral part of the development of a new drug prior to its approval, and strategies to manage these DDIs are routinely included in prescribing information.

Patients with infectious diseases in LICs are often predisposed to potential DDIs. Today, effective treatment of HIV, TB, or malaria frequently includes two or more drug molecules with diverse mechanisms of actions. Coinfection (e.g., TB in patients with HIV) and concomitant noninfectious disease, particularly with an aging population, undoubtedly requires the use of additional drugs, increasing the potential for DDIs. Despite recognition of DDIs by drug developers and regulators, management of DDIs and education of healthcare providers to ensure safe and effective use of anti‐infectives in LICs has not gained much attention. Although this is an area requiring significant consideration, there is currently a paucity of data available regarding optimal anti‐infective use in these patients and typically significant delays in between revisions of dosage guidelines. Using TB infection to illustrate, three central aspects regarding the identification and management of DDIs in LICs will be reviewed: (i) the DDI potential of anti‐infectives from pharmacological standpoints, (ii) the potential barriers to effectively manage DDIs in the LIC setting, including challenges with coinfection and comedication, and (iii) areas for future research so that optimal treatment at the individual patient level can be achieved.

## Pharmacokinetic DDIs—determination and current regulatory expectations

There are two main categories of DDIs—pharmacodynamics (PDs) and pharmacokinetics (PKs). In general, PD‐DDIs occur when the clinical effect of the victim drug is changed by the perpetrator, whereas PK‐DDIs result from modulation of one or more absorption, and disposition processes of the victim drug by a perpetrator drug. Once characterized, PK‐DDIs are often able to be managed effectively through methods such as changes in dosage or timing of administration. Only PK‐DDIs will be discussed here.

Current regulatory guidance requires testing for possible DDIs in early drug development for any new molecular entity (NME).^4^, ^5^ The testing is typically completed through a combination of *in vitro*,* in vivo*, and *in silico* studies to identify the metabolic and/or transport pathways susceptible to inhibition or induction, and to quantify the magnitude of interaction. Index substrates and inhibitors/inducers are used in clinical DDI studies (for evaluation of the NME as an inhibitor/inducer and substrate, respectively) to prospectively determine mechanistic interactions, as these compounds usually have a predictable change in exposure and the metabolic/transport pathways involved are well documented. Clinical studies can also be completed with medications commonly coadministered in the target population to determine DDI potential between comedications and the NMEs. Besides these standalone prospective DDI studies, one can assess DDI potential by collecting sparse samples from a nested study within a large trial (phase II or phase III) and use population PK (PopPK) modeling to analyze data obtained from the study. If adequately designed, PopPK analyses can help “characterize the clinical DDI and determine recommendations for dose modifications when investigational drug is a substrate.”^4^

Because clinical DDI studies may have limitations to inform untested clinical scenarios, such as the effect of dose regimens or of an inhibitor/inducer with different interaction potency, major regulators recommend the use of *in silico* methods, such as physiologically based pharmacokinetic (PBPK) modeling and simulations to complement the overall DDI assessment.^4^ A PBPK model combines physiological knowledge of the target population and drug characteristics (e.g., PK, physiochemical, absorption, and disposition properties) to define the PK of the drug.^6^, ^7^ The development of sophisticated models allows for the simulation of PK changes under various clinical scenarios by incorporating multiple interaction mechanisms and effects of multiple patient factors.^8^

Depending on the confidence of use, predictions using PBPK can be used in lieu of additional clinical DDI studies to support product labeling.^9^ For example, prediction of the effect of moderate or weak perpetrators may replace dedicated clinical DDI studies, provided that the PBPK model is verified with clinical PK data and information from dedicated DDI studies that used strong index inhibitors/inducers (drugs that increase or decrease the area under the curve (AUC) of a sensitive substrate ≥ five fold).^4^ PBPK models can also be used to research into DDI risk for previously understudied populations, such as pregnant women and children. Development of physiological models representative of these populations is critical for quantitative assessment of DDIs in these populations.^10^, ^11^, ^12^ It has to be recognized that application of PBPK is limited by the availability of data for both the drug (such as permeability and transporter involvement) and the population.

## Hurdles to effective management of DDI in LICs

Effectively mitigating the risk of PK‐DDIs in LICs has unique challenges that are not applicable in other regions, and there are important caveats to directly translating the findings of clinical studies to practical execution in these regions. In all regions, combination therapy is increasingly the mainstream strategy for anti‐infectives and treatments for HIV/AIDS (differentiated here from anti‐infective drugs for descriptive and comparative purposes). One advantage is to use more than two drugs with different mechanisms of action to synergistically combat a pathogen. However, many of these drugs are designed and evaluated from a monotherapy standpoint, as opposed to combination therapy as they will be used clinically. Furthermore, unlike the development of monotherapy, whose safety and efficacy in humans can be tested at various dose levels, development of combinations may be limited by the permutation of different doses for each partner drug in clinical trials, making it challenging to adequately determine full PK‐DDI and PD‐DDI potentials and to select optimal doses.

Beyond the issues that are faced in managing DDIs in all regions, the interpretation of PK‐DDIs in LICs is complicated by the current lack of understanding of the effects of comorbidity and other intrinsic factors. For example, malnutrition may affect the PK of test drug(s), and a patient with coinfection may take medications in addition to those for one target infection.^13^, ^14^ In conventional drug development, the understanding of PK‐DDIs is often based on studies completed in healthy adult volunteers. Although this is typically the most straightforward approach, as the contribution from other factors is minimal, these results may not be applicable in determining DDIs in target populations in LICs. Compared with healthy subjects, patients can display changes in drug disposition that are unexpected due to factors associated with infection, such as dehydration and changes in gastrointestinal motility from diarrhea and disease‐progression–dependent drug disposition (e.g., altered metabolism and, therefore, clearance). Subsequently, the response to DDIs can differ.

## Current understanding of DDIs associated with the world health organization‐recommended treatment for tuberculosis

As mentioned earlier, the use of multiple medications is commonplace for the treatment of major infectious diseases and co‐infection requires that medications indicated for different infections can be used concomitantly. Metabolism‐based and transport‐based DDIs among concomitant medications may lead to increased or decreased drug exposure, putting patients at risks of adverse events, loss of efficacy, and drug resistance. This section reviews the groups of drugs recommended for treatment of TB in LICs and potential PK‐DDIs among partner drugs in cases of common coinfections, such as with HIV, according to known or suspected mechanisms. For the latter, we used the University of Washington Metabolism and Transport Drug Interaction Database (www.druginteractioninfo.org).

### DDI potentials of TB drugs

With over 10 million new cases in 2017, most occurring in LICs, TB is one of the leading causes of death worldwide.^15^ For treatment of drug‐susceptible TB, the World Health Organization (WHO) recommends a 6‐month course of antibiotics—2 months of daily isoniazid (H), rifampin (R), pyrazinamide (Z), and ethambutol (E), followed by 4 months of isoniazid and rifampin, referred to as 2HRZE/4HR. A fixed‐dose combination of these anti‐TB drugs is recommended as compliance is higher than with separate drugs.^16^ Dosing strategies and PK properties pertaining to metabolism‐mediated and transport‐mediated DDI potential for these anti‐TB partner drugs are summarized in **Table** 1. From a delivery standpoint, current treatment guidelines from the WHO seem to focus on convenience and compliance. Obviously, the one‐dose‐fits‐all approach offers a simple solution for delivery, and the use of fixed‐dose combinations allows all medications to be taken on schedule to ensure compliance. However, these may not fit the needs of the individual patient and can limit the dose adjustments available to minimize the risk of PK‐DDIs.

**Table 1:** Summary of dosing strategies and PK properties pertaining to metabolism and transport‐mediated DDI potential for WHO‐recommended treatments for TB infection

Drug	Dose and range (mg/kg body weight)^13^	Dosing order/duration	Primary elimination route	*In vivo* metabolism/transport^a^		
				Substrate	Inhibitor	Inducer
Ethambutol	15 (15–20)	1st, 2 months	Renal	—	—	Not available
Isoniazid	5 (4–6)	1st, 2 months 2nd, 4 months	Renal	NAT2	CYPs 3A4, 2C19, 2E1, 1A2	CYP2E1
Pyrazinamide	25 (20–30)	1st, 2 months	Renal	Pyrazinamidase	—	Not available
Rifampicin	10 (8–12)	1st, 2 months 2nd, 4 months	Biliary	Arylacetamide deacetylase OATP1B1/1B3, P‐gp	OATP1B/3, P‐gp	CYPs 3A, 2B6, 2C9, 2C19, 2C8, 1A2 P‐gp

CYP, cytochrome P450; DDI, drug–drug interaction; NAT2, *N *‐acetyltransferase 2; OATP, organic anion transporting peptide; P‐gp, P‐glycoprotein; PK, pharmacokinetic; TB, tuberculosis; WHO, World Health Organization.

a Listed from most to least sensitive substrate (Michaelis constant (*K*_m_)), potent inhibitor (inhibitor constant (*K*_i_) or half‐maximal inhibitory concentration (IC_50_)), or inducer (fold‐increase enzyme activity).

As victim drugs, with the exception of rifampin, the recommended drugs are primarily renally cleared with metabolism occurring by non–cytochrome P450 (CYP) enzymes; therefore, DDI potential due to CYP modulation is not a primary concern. However, some of these drugs are substrates of polymorphic non‐CYP enzymes and/or transporters, and therefore, the PK of these drugs is likely to be altered in patients with impaired enzyme/transporter functions. For example, *in vitro* studies suggest that rifampin is a substrate of the major hepatic uptake transporters organic anion transporting peptides (OATPs), with *K*_m_ values of 1.5 μM^17^ and 2.3 μM^18^, ^19^ for OATP1B1 and OATP1B3, respectively. With chronic treatment of rifampin in patients with TB (median daily dose of 15.8 mg/kg) the exposure to rifampin was 3.16‐fold and 2.88‐fold higher in patients homozygous (*N* = 77) and heterozygous (*N* = 34) for the 388A>G mutation in *SLCO1B1*, the gene encoding the OATP1B1 transporter, respectively, compared with the reference group (*SLCO1B1* 388A/388A, *N* = 2).^20^

Similarly, isoniazid is mainly metabolized by the polymorphic enzyme *N*‐acetyltransferase 2 (NAT2). After administration of a 300 mg single dose of isoniazid in healthy subjects, the AUC of isoniazid was more than fourfold higher in NAT2 poor metabolizers (PMs; NAT2*5D/*7A and *6B/*6B) compared with normal metabolizers (NMs; NAT2*4/*4).^21^ A similar exposure increase was observed in NAT2 PM patients with TB compared with NM patients with TB who were on chronic treatment (5 mg/kg daily).^22^ With a significant fraction of individuals in LICs showing a PM phenotype (~ 33% of individuals in Sub‐Saharan Africa^23^ and 48% of Senegalese^24^, for example) significant increases in exposure are a common treatment concern for TB in these regions.

The first‐line TB partner drugs are inhibitors and inducers of many CYP enzymes and transporters, making them common perpetrators of DDIs (**Table** 1). To illustrate, rifampin is a known perpetrator of many interactions and is a recommended *in vitro* and clinical index inducer of multiple CYPs and an inhibitor of OATP1B1/1B3 by the US Food and Drug Administration.^25^ As an inducer, rifampin affects many CYP enzymes (e.g., CYPs 1A2, 2B6, 2C8, 2C9, 2C19, and 3A4), phase II enzymes, such as uridine 5′‐diphospho‐glucuronosyltransferases, as well as transporters, such as P‐glycoprotein (P‐gp); whereas as an inhibitor, rifampin primarily affects OATP1B1/1B3.^4^, ^5^, ^25^ With this broad scope of potential interactions, rifampin has been extensively studied and a wealth of *in vitro* and clinical DDI data are available. This prompted us to use rifampin to investigate the current state of DDI evaluation in LICs.

A search of the Drug Interaction Database yielded over 1,600 DDI studies (*in vitro* and clinical) involving rifampin. *In vitro*, there were 664 studies using rifampin as the perpetrator (inhibition or induction), with 85% concluding in a positive DDI. Similarly, rifampin has been predominantly studied clinically as a perpetrator, with 96.3% of the 1,007 clinical DDI studies using rifampin as an inhibitor and 87.9% concluding a positive DDI (defined by the US Food and Drug Administration as changes in victim exposure ≥ 25%).^4^ Although study design (i.e., frequency and magnitude of dose) can affect the observed changes in victim exposure and subsequent conclusions, the studies identified in the query almost exclusively utilized a 600 mg oral dose (single administration to evaluate inhibition and multiple doses for induction).

**Figure 1:**
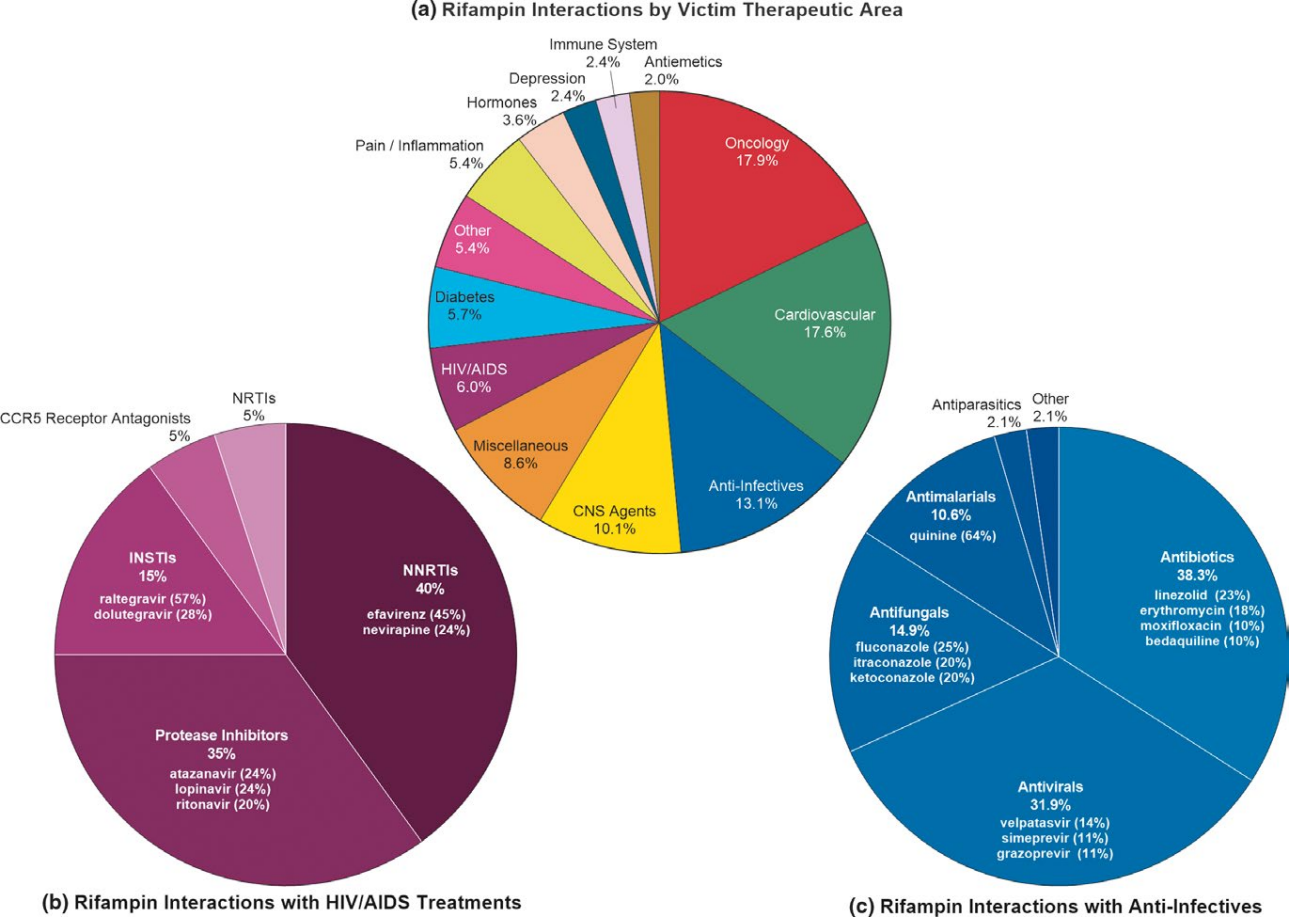
Therapeutic areas for reported drug–drug interactions (DDI)s with rifampin. Distribution of therapeutic areas with reported interactions (≥ 25% change in victim area under the curve (AUC)) with rifampin as the perpetrator for both induction and inhibition studies. Data were retrieved from the University of Washington Metabolism and Transport Drug Interaction Database on or before December 18, 2018. (**a**) The “miscellaneous” group includes drug categories, such as endogenous compounds and cannabinoids; “other” is a summation of the following therapeutic areas with less than five compounds tested (% of total): respiratory agents (1.9%), skin agents (1.2%), erectile dysfunction treatments (1.2%), dietary supplements and vitamins (0.6%), Parkinson's disease treatments (0.6%), antigout and uricosuric agents (0.5%), drug addiction treatments (0.5%), beta3‐adrenoreceptor agonist (0.5%), osteoporosis treatment (0.5%), muscle relaxants (0.5%), and migraine treatments (0.5%). (**b,c**). Interactions with rifampin by selected therapeutic area. Percentage indicates the relative contribution by that class to the overall number of observed interactions; the contribution from the primary drugs evaluated is included in parenthesis for the fractional contribution to interactions for that class. CCR5, C‐C chemokine receptor type 5; CNS, central nervous system; INSTI, HIV‐integrase strand transfer inhibitor; NRTI, nucleoside reverse‐transcriptase inhibitor; NNRTI, non‐nucleoside reverse‐transcriptase inhibitor; RIF, rifampin.

Clinical DDI studies using rifampin as the perpetrator were completed for a broader range of compounds than *in vitro* and included over 300 different compounds as victims (113 substrates were evaluated *in vitro*, and only 37 of those compounds were evaluated both *in vitro* and *in vivo*). These include 294 (83%) victims of induction, 24 (7%) for inhibition, and 35 (10%) for both. Identified interactions ranged from a 99.7% decrease in victim AUC (induction, rifampin—budesonide^26^) to an increase in victim AUC of almost 1,400% (inhibition, rifampin—asunaprevir^27^). In addition to the wide range in the magnitude of interactions, these studies were performed for drugs in 90 classes from 24 diverse therapeutic areas (**Figure** 1 **a**) illustrating the high potential for DDIs during TB treatment. Two therapeutic areas were investigated in more detail, anti‐infectives and HIV/AIDS treatments, as coinfection is common in LICs. Investigation of potential DDIs between rifampin and these common treatment classes for coinfection account for 19% of identified studies—10.7% between rifampin and anti‐infectives and 7.2% with treatments for HIV/AIDS (**Figure** 1 **b,c**).

### DDI under coinfection

In individuals living with HIV, coinfection with TB is ~ 20 times more likely compared with those without HIV. In fact, just over half of the reported cases of TB in 2017 were in people living with HIV, most of who were already on antiretroviral therapy.^15^ Of the four preferred first‐line treatments for HIV—efavirenz, emtricitabine, lamivudine, and tenofovir disoproxil fumarate—all are substrates of multiple enzymes and transporters that are inhibited and/or induced by rifampin. These drugs are not the exception, as the alternative first‐line treatments, such as dolutegravir and nevirapine, are also substrates of enzymes and transporters that can be affected by rifampin. In fact, higher dosages of dolutegravir are recommended in those taking concomitant rifampin to ensure adequate plasma concentrations.^28^ Among the first‐line HIV medications, efavirenz is most susceptible to interactions because it is a substrate of CYP2B6, an enzyme that is not only polymorphic but also inducible by rifampin. When studied in healthy volunteers, repeated dosing of rifampin (450 mg/day for 7 days) significantly decreased the AUC of efavirenz (38.6%).^29^ This result has also been observed in multiple other studies completed in healthy volunteers, with a significant decrease in efavirenz exposure by coadministration with rifampin (range: 17.8–61.0% decrease),^30^ and in all cases, the decreased exposure is attributed to induction of CYP2B6 by rifampin (half‐maximal effective concentration = 0.127 μM).^31^ With the observed decreases in exposure, it is recommended to increase efavirenz dose in adult patients who receive concurrent rifampin to ensure that adequate plasma concentrations are achieved.^32^ Low‐dose (400 mg) efavirenz has been conditionally approved as treatment by the WHO to reduce the occurrence of adverse events; however, the PK and efficacy in patients also receiving TB treatment has yet to be determined.^28^

However, interpretation of the results from a healthy volunteer study for efavirenz is highly complicated. First, CYP2B6 is polymorphic, and efavirenz itself can induce multiple drug metabolizing enzymes (CYPs and uridine 5′‐diphospho‐glucuronosyltransferases). Indeed, > 20 clinical studies with chronic efavirenz treatment (600 mg daily) in patients have shown substantial increases of 2‐fold to 4‐fold in the exposure of efavirenz in CYP2B6 PM or intermediate metabolizers compared with patients with normal CYP2B6 function, with one study showing a 44‐fold increase in the AUC of efavirenz in CYP2B6 PMs (*CYP2B6* 516T/516T, *N* = 2) compared with NMs.^33^ Without genotyping prior to initiating treatment, it is possible that patients may have significantly higher exposure to efavirenz than expected, which further increases their risk for undesirable events—both as a victim of interactions and as a perpetrator of comedications. Second, decreases in efavirenz exposure induced by coadministration with rifampin in healthy subjects do not manifest in patients coinfected with TB and HIV. In one study, efavirenz clearance decreased by 29.8% in patients coinfected with TB and HIV receiving dual treatment, as compared to those only receiving HIV treatment.^34^ In other studies, nonsignificant but variable changes in efavirenz AUC, ranging from a 6.7% decrease to a 1.9% increase, have been reported.^35^ This highlights that coinfection, comedication, and other intrinsic factors unique to patients together can change the PK of both the perpetrator and victim drugs, leading to unique interactions or magnitudes of interactions that cannot be readily extrapolated from findings in healthy subjects.

Similar to HIV, coinfection of TB with malaria is common in LICs and the recommended treatments for malaria show a high susceptibility to DDIs.^36^ In fact, studies have shown that induction of metabolic pathways by rifampin causes significant decreases in drug exposure for the primary therapies, including artemether, lumefantrine, mefloquine, and quinine.^30^, ^36^ These changes can cause treatment failure as the systemic concentrations are below the level needed to fully combat the infection. To illustrate, in healthy volunteers, concomitant administration of rifampin significantly increased mefloquine metabolism (281% increase in clearance) reducing plasma concentrations of mefloquine by 67.9%.^37^ In patients with uncomplicated falciparum malaria treated with quinine, the addition of rifampin resulted in a 75.4% decrease in quinine AUC and 18.1% decrease in peak plasma concentration (C_max_). This decrease in quinine concentrations was associated with a fivefold increase in reinfection compared with those patients only receiving quinine. These significant changes in exposure and subsequent treatment failures can be attributed to induction of CYP3A, evidenced by significantly higher metabolite exposure (five fold increase) in those taking rifampin.^38^

As another example, rifampin caused significant decreases in exposure for partner drugs in Coartem® (a fixed‐dose combination including artemether and lumefantrine; Novartis, East Hanover, NJ) administered in an HIV‐positive Ugandan population without comorbid malaria. In these patients, the AUC of both artemether and lumefantrine were significantly decreased in the presence of rifampin (89% and 68% decreases, respectively).^39^ This is again consistent with induction of enzymes, such as CYP2B6 and CYP3A4 and possibly the induction of intestinal efflux transporters, such as P‐gp, resulting in lower absorption of the drugs.^39^ These findings imply that patients with TB and malaria coinfection may require higher doses of antimalarial drugs that are susceptible for DDIs resulting from induction of P‐gp and/or metabolizing enzymes.

### Disease effect on drug PK

Concerns in coinfected populations are not solely limited to interactions between treatments for the infections, however. It cannot be assumed that the PK of either the victim or perpetrator compounds is consistent among healthy subjects, singly infected, and coinfected patients. A study conducted in patients coinfected with TB/HIV in Burkina Faso found that although rifampin exposure was increased when it was used in a combined therapy, with the mechanism for the increase still unknown but likely due to increased absorption and/or decreased clearance due to liver toxicity caused by drugs, such as nevirapine, systemic concentrations of rifampin still remained markedly lower than in other populations.^40^ In fact, no subjects in the study had sustained plasma levels of rifampin above the accepted therapeutic threshold of 8 μg/mL after 10 weeks of standard dosing (10 mg/kg/day).

This unexplained decrease in exposure is not exclusive to rifampin. A similar decrease in exposure in coinfected patients compared with those with only TB infection has also been observed for isoniazid (C_max_ = 11 μg/mL in patients with TB compared with 7.0 μg/mL in patients with TB/HIV).^41^ For many anti‐infectives, threshold concentrations must be reached for effective treatment, and significant decreases in exposure, such as these, may lead to an increased risk for treatment failure and, more importantly, an increased risk of developing drug resistance. Conversely, quinine also shows disease‐dependent changes in PK, with clearance in patients with malaria being significantly decreased compared with healthy subjects.^42^ Although the exact mechanisms for these disease‐related changes are unknown, it is likely that changes in absorption, protein binding, and altered hepatic function can all contribute to changes in systemic concentrations.^42^, ^43^ These changes in exposure are still unable to be accurately predicted due to the number of covariates present, resulting in a unique challenge not only in ensuring effective treatment but also in accurately determining risk and developing strategies to mitigate potential DDIs.

### Target global health populations

Beyond the inherent complexities of treatment of coinfections, the understanding and evaluation of DDIs in LICs is further complicated by the occurrence of infection and co‐infection in specific populations, such as children, pregnant women, and women on oral contraceptives. Conventionally considered as special populations in mainstream drug development, these populations in fact are target populations of product development in global health. Due to the paucity of data on both the expected PK and expected magnitude of DDIs in these patients from the inherent difficulties in conducting clinical research to collect such data, the potential risk almost always has to be extrapolated from healthy, nonpregnant adults in order to optimize dose selection. Such extrapolation is not straightforward and is challenged by the lack of quantitative understanding of the unique physiology of these patients that may impact the PK and PD characteristics of both victim and perpetrator drugs. For example, a recent study comparing the AUC fold‐change of 24 drug pairs in adult and pediatric patients showed that, more often than not, there was a significantly different magnitude of effect between the two groups (69.7% of pediatric studies were > 1.25‐fold or < 0.8‐fold of the adult values).^44^ Research is needed to systematically understand such differences.

Treatment of TB in pregnant women is also not immune from this imbalance in research. Currently, the WHO does not recommend any changes in treatment protocol for pregnant women.^16^ Although first‐line treatments are currently considered safe for both mother and fetus, there is little research supporting this and at least three medications—isoniazid, rifampin, and ethambutol—are able to cross the placental barrier and are known to have an increased risk for adverse events, such as hepatotoxicity.^45^ Research on treatment of TB in children also lags behind that of adults in LICs. Historically, a combined approach of 2HRZE/4HR, the same as what is preferred with adults, has been used to treat TB in children without appreciating developmental and ontogeny changes. Whereas the 2014 WHO guidance on treatment of TB in children did propose updated daily doses, these changes are based primarily on observational studies and “moderate‐quality” evidence.^46^, ^47^ Although dose modification could help to ensure that sufficient concentrations of drug are reached, there is little evidence on the safety of these doses in children and limited understanding of the potential for hepatotoxicity. These populations become more complex when coinfection exists. It is estimated that almost half of adult HIV‐related TB deaths in 2017 were in women of childbearing age.^48^ Pregnant women with concurrent TB/HIV infection face higher risk of poor delivery outcomes and higher mortality rates.^49^, ^50^ Coinfection and subsequent comedication increase the potential for drug interactions far beyond what is predicted for nonpregnant adults due to the physiological changes during pregnancy that can dramatically affect drug exposures in both pregnant women and fetuses.

Children also bear the burden of coinfection with over 50,000 TB‐related deaths in 2016 occurring in children living with HIV.^50^ In children, the relationships between exposure and toxicity as well as effective dosing for anti‐HIV treatments, especially when combined with TB medications, such as rifampin, are still unknown. To illustrate, in children under the age of 3 years old who were cotreated for HIV/TB infection, nevirapine exposure was 41% lower compared with those without TB.^51^ Additionally, nevirapine concentrations are more variable than those seen in adults, which makes prediction of interactions much more difficult. Similar changes in exposure have also been found for rifabutin, a rifampin alternative. In a clinical study evaluating the PK and safety of rifabutin in children also on anti‐HIV treatment, severe neutropenia was observed for all subjects resulting in the early termination of the study.^52^ It was found that concentrations of rifabutin were more than twofold higher than those found in adult studies, which could be attributed to decreased CYP3A metabolism due to immature enzyme function and inhibition by ritonavir (HIV treatment).

Similarly, DDIs with oral contraceptives has been a growing topic of research in recent years as oral contraceptives rely on a minimum concentration for efficacy and changes in exposure can result in treatment failure.^53^ For example, coadministration of rifampin and dienogest/estradiol valerate resulted in an 83% decrease in dienogest AUC and 44% decrease in the AUC of estradiol. This resulted in concentrations falling below the minimum effective concentrations leading to an increased risk for unplanned pregnancies.^54^

## Future directions

The challenges and hurdles described in the previous sections call for the need to establish a quantitative understanding of relevant population‐specific attributes and drug‐related properties for effective assessment of DDIs in target populations of LICs. Encouragingly, research into drug disposition and interactions in special populations has increased in recent years, which has led to enhanced understanding of the physiological component of a PBPK model for these populations (virtual populations).^10^, ^55^, ^56^, ^57^ For example, a dedicated guidance on diagnosis and management of TB in children was released by the WHO in 2014, marking the first departure from a uniform treatment approach for adults and children. Here, dosage recommendations could be updated to reflect age‐specific predictions for exposure based on enzyme ontogeny and accumulated clinical data in those under the age of 10 years old.^47^ Despite such advances, large gaps still remain in the fundamental understanding of many population specific variables, especially for those in LICs.

To fully understand the current landscape, existing DDI studies with comedications for relevant anti‐infective and HIV/AIDs drugs first need to be evaluated in detail to identify specific areas requiring further investigation. A panel of outcome measures, such as PK parameters for drug and metabolite, major PD end points, and safety observations should be evaluated and compiled. Furthermore, information on the inherent changes in patient physiology from these diseases that cannot be captured from healthy volunteers should be collected when available. These observations can be further refined using *in vitro* techniques when appropriate to determine the mechanism(s) behind the changes. The understanding of relevant population‐related and drug‐related properties, through the compilation of clinical data and *in vitro* research, enables the mechanistic prediction of potential DDIs through predictive models, such as PBPK. Through modeling, preliminary predictions can be made for a specific population under various clinical scenarios, such as potential drug combinations, allowing for clinical studies to be prioritized so that resources are allocated to the most needed studies. This knowledge will also allow for the design of appropriate protocols that fit the needs of the community best—ideally reducing the duration of the study and optimizing patient follow‐up requirements. Depending on the confidence level of PBPK models, simulations can be used to support dosing recommendations in scenarios that cannot be informed by the conduct of clinical DDI studies, due to either ethical reasons or feasibility considerations. Understanding these DDIs is only part of the solution, however. The knowledge gained on these topics will then need to be translated to strategies that can be implemented in LIC communities. Cooperative efforts in manufacturing additional dosage options and updated training for healthcare providers will need to be undertaken to ensure that the benefits from the ability to understand and predict DDIs are available to those who are at risk.

Further research into these areas will also serve to complement related, ongoing efforts within the scientific community. For example, Lesko *et al*.^58^ recently proposed the collaboration of multidisciplinary research to evaluate oral contraceptive‐based DDIs. Indeed, the combination of PBPK modeling and model‐based meta‐analysis allows for an integrated approach to identifying and subsequently bridging existing knowledge gaps within this field. Additionally, collaborative efforts to develop PBPK models for antimalarial drugs and anti‐TB drugs are currently underway between The Bill & Melinda Gates Foundation and organizations, such as Medicines for Malaria Venture and the Critical Path Institute. Together, these research activities enable researchers to capitalize on the existing and emerging knowledge in this field and allow the utilization of modeling and simulation methods to assess and manage complex DDIs in target populations of LICs.

Although significant progress has been made to better understand PK‐DDIs in LICs, there is still work to be done. Better understanding the underlying conditions and the resultant changes in drug disposition in these populations will allow for the development of effective risk mitigation strategies when comedication is required. Acquiring a mechanistic understanding of the unique confounding factors in these patients, as well as the application of PopPK techniques to identify critical covariates, is essential for effectively informing and implementing predictive models to evaluate these interactions and for the progression to the implementation of population‐specific treatment strategies.

## Funding

This work was supported by a grant from the Bill and Melinda Gates Foundation (OPP1199811).

## Conflict of Interest

The authors declared no competing interests for this work.
